# A transitional care program in a technologically monitored in‐hospital facility reduces the length of hospital stay and improves multidimensional frailty in older patients: a Randomized Clinical Trial

**DOI:** 10.1007/s40520-024-02821-8

**Published:** 2024-08-06

**Authors:** Alberto Pilotto, Wanda Morganti, Marina Barbagelata, Emanuele Seminerio, Simona Morelli, Romina Custureri, Simone Dini, Barbara Senesi, Camilla Prete, Gianluca Puleo, Carlo Berutti Bergotto, Francesco Vallone, Carlo Custodero, Antonio Camurri, The PRO‐HOME Project Investigators Group

**Affiliations:** 1 Department of Geriatric Care, Neurology and Rehabilitation, E.O. Galliera Hospital, Mura delle Cappuccine 14, 16128 Genoa, Italy; 2 Department of Interdisciplinary Medicine, University of Bari “Aldo Moro”, Bari, Italy; 3 Informatic and Technology Unit, E.O. Galliera Hospital, Genoa, Italy; 4 Casa Paganini‐InfoMus Research Center, Department of Informatics, Bioengineering, Robotics and Systems’ Engineering (DIBRIS), , University of Genova, Genoa, Italy

**Keywords:** Transitional care program, Multidimensional frailty, Multidimensional Prognostic Index, Comprehensive Geriatric Assessment

## Abstract

**Background:**

Longer length of hospital stay (LOS) negatively affects the organizational efficiency of public health systems and both clinical and functional aspects of older patients. Data on the effects of transitional care programs based on multicomponent interventions to reduce LOS of older patients are scarce and controversial.

**Aims:**

The PRO‐HOME study aimed to assess the efficacy in reducing LOS of a transitional care program involving a multicomponent intervention inside a technologically monitored in‐hospital discharge facility.

**Methods:**

This is a Randomized Clinical Trial on 60 patients (≥65 years), deemed stable and dischargeable from the Acute Geriatrics Unit, equally assigned to the Control Group (CG) or Intervention Group (IG). The latter underwent a multicomponent intervention including lifestyle educational program, cognitive and physical training. At baseline, multidimensional frailty according to the Multidimensional Prognostic Index (MPI), and Health‐Related Quality of Life (HRQOL) were assessed in both groups, along with physical capacities for the IG. Enrolled subjects were evaluated after 6 months of follow‐up to assess multidimensional frailty, HRQOL, and re‐hospitalization, institutionalization, and death rates.

**Results:**

The IG showed a significant 2‐day reduction in LOS (median days IG = 2 (2–3) vs. CG = 4 (3–6); *p* < 0.001) and an improvement in multidimensional frailty at 6 months compared to CG (median score IG = 0.25(0.25–0.36) vs. CG = 0.38(0.31–0.45); *p* = 0.040). No differences were found between the two groups in HRQOL, and re‐hospitalization, institutionalization, and death rates.

**Discussion:**

Multidimensional frailty is a reversible condition that can be improved by reduced LOS.

**Conclusions:**

The PRO‐HOME transitional care program reduces LOS and multidimensional frailty in hospitalized older patients.

*Trial registration*: ClinicalTrials.gov n. NCT06227923 (retrospectively registered on 29/01/2024).

## Background

Prolonged length of hospital stay (LOS) has detrimental effects at both individual level, being associated to the loss of some independence in basic activities of daily living (ADL) [1] and increased insurgence of patients’ complications [2] and at healthcare system level determining increased costs related to the unnecessary occupation of hospital beds [3]. Indeed, 8% of hospitalized older patients experiences a mean of 1.4 days longer hospital stay than young‐adult patients [4], although in many cases they could be considered dischargeable, presenting clinically stable conditions and not needing further diagnostic or therapeutic procedures [3]. The main causes of delay in discharging are the onset of new disabilities, difficulties in arranging home‐care assistance or admission to long‐term care facilities [5], and an overall deficiency of social and health resources to address their post‐acute care requirements [6].

A recent review suggests that in the general population, strategies such as early discharge planning, transitional care programs (TCP), and multidisciplinary care could be effective in reducing LOS [7]. However, data for high‐risk older patients are scarce and controversial [7].

In counteracting the postponement of hospital discharge, TCPs based on a Comprehensive Geriatric Assessment (CGA) seem to be the most effective [8]. Indeed, the CGA‐based multidimensional approach is the optimal model to evaluate the medical, functional and psychosocial needs of older people and develop a personalized intervention to reduce several negative outcomes (e.g., mortality, institutionalization, risk of delirium) in different settings (e.g., medical and surgical clinics and wards, emergency department) [9–12].

In this context, the development and application of information and communication technologies (ICT), assistive technologies, and human‐computer interaction technologies opened new avenues for monitoring health parameters and tracking clinical disorders in older adults [13]. Building e‐health technologies has started to emerge as a potential remedy for the structural weakness of the family‐ and private‐based care systems as well as the limited capacity of the public healthcare systems [14].

To overcome these organizational issues, technological monitoring (telemonitoring) could help enhance quality of care, independence in daily living, and safety. Ambient Assisted Living (AAL) and home monitoring systems aid healthcare professionals and caregivers in offering care to older adults by potentially prevent acute events and early detect adverse events and emergencies, such as falls [15–18].

Given this background and inspired by the growing evidence suggesting the effectiveness of multicomponent CGA‐based intervention in improving health outcomes in hospitalized older adults [9, 10], we tested the effect of a multicomponent intervention (namely PRO‐HOME) in reducing LOS and eliciting a change in multidimensional frailty in older patients, employing a telemonitoring system inside a protected in‐hospital facility.

## Methods

### Study design and participants

The PRO‐HOME project (registered on clinicaltrials.gov: NCT06227923) is a Randomized Clinical Trial (RCT) aimed at testing the clinical and organizational effectiveness of a protected discharge care model based on a multicomponent intervention carried out inside an intra‐hospital environment equipped with monitoring technologies in hospitalized multimorbid and polytreated older people. PRO‐HOME is included in the MULTIPLAT_AGE Research Net‐Program which is a network project involving five different Italian partners, co‐funded by the Italian Ministry of Health and the Liguria region. The study was conducted in accordance with the Declaration of Helsinki and approved by both the Italian Ministry of Health (27/02/2018, No 44761) and the Local Ethical Committee (protocol n.176/2021).

Sample size calculation was established and reported elsewhere resulting in 60 subjects to be enrolled [19].

Sixty patients were consecutively enrolled at the Acute Geriatric Unit of the Galliera Hospital in Genoa (Italy) from May 2021 to April 2023. Inclusion criteria were subjects: (a) aged over 65 years, (b) admitted to the hospital for an acute event, (c) deemed stable and dischargeable, (d) with preserved functional abilities and personal autonomy (Activities of Daily Living [20] score ≥ 3/6), (e) with normal cognitive functioning or mild impairment (Short Portable Mental Status Questionnaire [21] score ≤ 5/10), (f) willing to participate in the study. Older people with behavioral disorders, psychotic symptoms, or moderate/severe impairments in functional autonomy and/or cognition were excluded.

Patients, after signing informed consent, were randomized (1:1 ratio) to the Intervention Group (IG) or the Control Group (CG) using a randomization sheet. Patients and the recruiting healthcare staff did not know the subjects’ group allocation until the assignation of a random alphanumeric code identifying the belonging to a specific group. Afterwards, thirty of them were included in the PRO‐HOME smart home facility (IG), whilst the CG patients remained in the hospital acute ward until discharge undergoing usual care.

### Intervention: the PRO‐HOME area and the multicomponent intervention

The IG while in the protected PRO‐HOME area, was monitored through smart devices (both wearable and environmental) and took part in a multicomponent intervention.

The smart devices are intended to guarantee the safety of the patients inside the PRO‐HOME area. In case of emergency, an operational emergency protocol is implemented by involved professionals. More details about the emergency procedures are described elsewhere [19].

Specifically, the smart home is equipped with Closed Circuit Televisions (CCTV) cameras and a depth infra‐red camera (Kinect Azure), a lifeline device with an emergency call button that can alert healthcare professionals in real‐time, a robotic device (PadBot P2) to carry a tablet to the patient for communication between patient/caregiver and health professionals, and a Fitbit Sense smartwatch to monitor clinical (heart rate, blood oxygenation, etc.) and physical parameters (number of steps taken, distance walked, etc.).

The architectural structure of the PRO‐HOME was made of one bedroom, a bathroom, a living room with a sofa‐bed which can allow, if wanted, the presence of a caregiver, and a large corridor where the Kinect was installed.

The multicomponent intervention addressed to the IG was based on physical and cognitive activation exercises, lifestyle educational program, and an optional music‐based relaxation activity [22]. All the planned activities were performed inside the PRO‐HOME area or in specific clinics inside the hospital, during the time spent in the smart‐home. The cognitive intervention stimulated all the principal cognitive domains (e.g., memory, attention) [23]. The educational program focused on giving suggestions about chronic conditions management, dietary habits, sleep quality enhancement, and appropriate use of drugs and hygiene care [24]. The physical activity was tailored to the patient’s assessment of functional performance and was carried out with the aid of a physical therapist. The patients were also trained for the optional use of a music‐therapy self‐administered protocol [25]. Further details on the PRO‐HOME study design, the ecosystem, technological set‐up, and the intervention protocol have been previously published elsewhere [19].

### Assessment protocol

Data about demographic characteristics (age, gender, education) and clinical information were recorded at admission. At baseline, all the participants underwent a standard CGA carried out by a team of healthcare professionals including geriatricians, nurses, and physiotherapists. From the CGA data, the Multidimensional Prognostic Index (MPI) [26] was calculated to assess multidimensional frailty; moreover, participants were evaluated for depression using the Geriatric Depression Scale (GDS‐15) [27], need for nursing care [28] and health‐related quality of life with the Health‐related Quality of Life Short‐Form‐12 (SF‐12) [29]. In the IG, participants were also assessed for levels of physical performance through the Short Physical Performance Battery (SPPB) [30], and the hand grip strength.

The study included three follow‐up remote visits after 1, 3, and 6 months from discharge in which information on multidimensional frailty using the TELE‐MPI, a validated telephone‐administered version of the MPI [31], the SF‐12, and registration of eventual negative health outcomes including hospitalization, institutionalization, and death was recorded. Standard MPI and TELE‐MPI have already shown strong agreement thus proving their interchangeability [31].

#### Multidimensional frailty

The Multidimensional Prognostic Index [26] is a widely used and validated CGA‐based tool for frailty assessment and identification of older patients’ prognosis.

It assesses different domains:

- functional status through the Basic—ADL [20]—and Instrumental Activities of Daily Living—IADL [32];
- mobility capacities/risk of developing sore pressures using the Exton Smith Scale—ESS [33];
- nutritional status through the Mini Nutritional Assessment—Short Form—MNA‐SF [34];
- cognitive status using the Short Portable Mental Status Questionnaire—SPMSQ [21];
- general health status using the Comorbidity Index of the Cumulative Illness Rating Scale—CIRS‐CI [35];
- polypharmacy counting the number of drugs regularly taken;
- cohabitation status.

The eight scales included are summarized using three risk score categories (0, 0.5, and 1) which are summed and then divided by the number of completed scales. The score obtained, which ranges from 0 to 1, can be in turn classified as low risk of frailty (MPI‐1, scores less than 0.33), moderate risk of frailty (MPI‐2, scores between 0.34 and 0.66), and high risk of frailty (MPI‐3, scores greater than 0.67). In this study, were used both the MPI standard version for hospitalized patients at baseline and the telephone‐administered version (TELE‐MPI) at follow‐ups [31]. The two versions differ in the mobility assessment which in the standard version is assessed by the Exton‐Smith scale whilst in the TELE‐MPI by using a modified Barthel Index mobility index (assessing capacities of walking, climbing stairs, and get in/out of bed).

#### Other measurements

The Geriatric Depression Scale‐15 (GDS‐15 [27]) is one of the most widely used rating scales for assessing depression in older people. It consists of 15 items with dichotomous answers, and its score ranges from 0 to 15. Scores between 5 and 8 suggest the presence of mild depression, from 9 to 11 can be classified as moderate depression, and scores of 12 or higher indicate severe depression.

The SF‐12 [29], derived from the longer version with 36 items, assesses health‐related quality of life (HRQOL) giving two different scores referred to physical (bodily pain, general health, etc.) and mental (vitality, social functioning, etc.) components (its summary scores range from 0 to 100). In older people samples, the mean scores for the physical component are between 40.69 and 45.53 whilst the mental component on average varies from 51.35 to 53.67.

The need for nursing care was assessed using a specific scale, extracted from the S.Va.M.A. [36] assessment report. It ranges from 0 (no need for nursing care) to 80 (maximum nursing assistance needed), investigating, for example, the need for devices for incontinence or parenteral nutrition.

Assessment of physical functioning included the measurement of upper limbs muscle strength with hand grip test strength measured by hydraulic manual dynamometer, walking speed over 4 m, and the score at the Short Physical Performance Battery (SPPB) a composite test evaluating balance, gait, and sit‐to‐stand ability [30].

### Statistical analysis

Continuous variables were summarized using medians and inter‐quartile range (IQR) or means (Ms) and standard deviations (SDs) if normally distributed, whilst frequencies and percentages were used for categorical variables. Using the Shapiro–Wilk test, each variable was tested for normality distribution. The two groups (IG and CG) were compared using a *t* test for normally distributed variables or Mann‐Whitney in case of violation. Categorical variables were compared using Chi‐square tests. The effect size was computed as Cohen’s d or rank‐biserial correlation coefficient. For longitudinal comparisons (baseline vs. follow‐ups, for both the two subsamples), Wilcoxon signed rank tests or *t* tests were used based on non‐normal or normal distributions. The distribution of the adverse health outcomes (institutionalizations, re‐hospitalizations, and deaths) was compared between IG and CG using a Chi‐square test.

All two‐tailed statistical tests were considered statistically significant with a *p* value of 0.05 or less. Jamovi and SPSS (version 21.0 for Mac) were used for the analyses.

## Results

### Baseline and at hospital discharge results

The initial sample consisted of 60 patients, equally randomized between the IG (30 patients) and the CG (30 patients). Two subjects were excluded from the analyses as outliers for MPI score (MPI mean 0.397 ± 0.117; both subjects were above more than 2 standard deviations).

Table 1 shows the demographic and functional characteristics of the two subgroups (IG and CG). The two groups differ only in the cohabitation status, i.e. the IG has fewer subjects institutionalized and living alone compared to the CG.

**Table 1:** Descriptive statistics and comparison of the two subgroups

Parameter (*n* = 58)	Intervention group (*n* = 30)	Control group (*n* = 28)	*p* value
Age (mean ± SD)	81.6 ± 7.2	83.4 ± 5.4	0.284^a^
Males gender (*n*, %)	18/30 (60.0%)	12/28 (42.9%)	0.192^b^
Education (*n* = 42) (median, IQR)	13 (8–13)	8 (5–13)	0.333
*MPI domains*			
ADL score (median, IQR)	6 (5–6)	5 (3–6)	0.072
IADL score (median, IQR)	5.5 (5–7.8)	4.5 (3–6.3)	0.082
ESS score (median, IQR)	19 (17–19)	18 (17–19.3)	0.987
SPMSQ score (median, IQR)	0 (0–2)	1 (0–2)	0.337
MNA‐SF score (median, IQR)	9.5 (7.25–11)	9 (7–10)	0.420
CIRS‐CI score (median, IQR)	4 (3–5)	4 (3–4)	0.107
Number of drugs (mean ± SD)	8.0 ± 3.1	6.8 ± 3.3	0.153^a^
*Cohabitation status (n, %)*			
With family	26/30 (86.7%)	17/28 (60.7%)	
Institutionalised	0/30 (0%)	3/28 (10.7%)	0.046*^,b^
Alone	4/30 (13.3%)	8/28 (28.6%)	
MPI score (median, IQR)	0.344 (0.313–0.438)	0.438 (0.313–0.453)	0.071
SF‐12—physical component (mean ± SD)	36.8 ± 7.6	34.0 ± 8.6	0.192^a^
SF‐12—mental component (mean ± SD)	46.2 ± 10.0	43.3 ± 10.0	0.272^a^
GDS‐15 (median, IQR)	4 (2–5.8)	4 (2.8–6)	0.888
Need for nursing care (*n* = 57) (median, IQR)	0 (0–5)	0 (0–5)	0.699
Hand grip (*n* = 30) (median, IQR)	22 (20–28)	N.A	N.A
Gait speed (*n* = 30) (mean ± SD)	0.67 (0.53–0.85)	N.A	N.A
Heart rate (median, IQR)	72.0 (68.3–80.0)	76.0 (68.8–80.3)	0.731

*MPI* Multidimensional Prognostic Index, *ADL* Activities of Daily Living, *IADL* Instrumental Activities of Daily Living, *ESS* Exton‐Smith Scale, *SPMSQ* Short Portable Mental State Questionnaire, *MNA–SF* Mini‐Nutritional Assessment–Short Form, *CIRS‐CI* Cumulative Illness Rating Scale—Comorbidity Index, *GDS‐15* Geriatric Depression Scale—15 items, *N.A.* not applicable

^*^ Statistically significant *p* value

^a^Comparison made using *t* test

^b^Comparison using Chi‐square test

Table 2 shows the clinical characteristics of patients with the prevalence of concomitant diseases as assessed by the CIRS, in the two groups. No significant differences were observed between the two groups.

**Table 2:** Distribution of diseases in the two subgroups and their comparison

Diseases	Intervention group (*n* = 30) (*n*, %)	Control group (*n* = 28) (*n*, %)	*p* value
Cardiac	20 (66.7%)	13 (46.4%)	0.120
Hypertension	17 (56.7%)	12 (42.9%)	0.293
Vascular	9 (30%)	7 (25%)	0.670
Respiratory	18 (60%)	10 (35.7%)	0.064
Eye/ear/nose/throat/larynx	2 (6.7%)	1 (3.6%)	0.595
Upper gastrointestinal	10 (33.3%)	11 (39.3%)	0.637
Lower gastrointestinal	9 (30%)	9 (32.1%)	0.860
Hepatic/biliary	2 (6.7%)	2 (7.1%)	0.943
Renal	3 (10%)	2 (7.1%)	0.698
Genitourinary	6 (20%)	11 (39.3%)	0.107
Musculoskeletal	10 (33.3%)	7 (25%)	0.486
Neurological	6 (20%)	4 (14.3%)	0.565
Endocrine/metabolic	12 (40%)	11 (39.3%)	0.956
Psychiatric	5 (16.7%)	4 (14.3%)	0.802

At discharge, patients of the IG showed a statistically lower LOS compared to patients in the CG (IG median (IQR) LOS = 2 days (2–3) vs. CG median (IQR) LOS = 4 days (3–6), Mann–Whitney’s *U* = 145; *p* < 0.001), with a 66% likelihood that the participant from the control group stayed longer in the hospital (rank‐biserial correlation coefficient = 0.66) (see Table 3). While IG participants included both in MPI‐1 and MPI‐2 subgroups stayed less in the hospital than the CG participants, however the MPI‐2 participants had a higher effect size (0.68) than the MPI‐1 participants (0.62), suggesting PRO‐HOME gave a greater benefit to frailer older people.

**Table 3:** Statistics of LOS for the two subgroups (IG and CG)

Length of stay	Intervention group (*n* = 30)	Control group (*n* = 28)	*p* value	Effect size
*Total sample*				
Median (IQR)	2 (2–3)	4 (3–6)	<0.001*	0.66
MPI‐1 (*n* = 23)	2 (1–4)	4 (1–6)	0.016*	0.62
MPI‐2 (*n* = 35)	2 (1–3)	4 (1–10)	<0.001*	0.68

*MPI* Multidimensional Prognostic Index

^*^ Statistically significant *p* value

### Six‐month follow‐up

At the 6‐month follow‐up assessment (see Table 4), information about the adverse health outcomes (institutionalization, re‐hospitalization, and death) was gathered for all the 58 patients (30 in the IG and 28 in the CG). More specifically, 5 patients were institutionalized (4 in the CG and 1 in the IG; *χ*^2^ = 2.21; *p* = 0.138), 4 were re‐hospitalized (1 in the CG and 3 in the IG; *χ*^2^ = 0.93; *p* = 0.334), and 1 died (1 in the IG; *χ*^2^ = 0.95; *p* = 0.330).

**Table 4:** Descriptive statistics at the second follow‐up (after 6 months from discharge)

Parameter	Intervention group (*n* = 30)	Control Group (*n* = 28)	*p* value
*MPI domains*			
ADL score (*n* = 45) (median, IQR)	6 (5–6)	5 (4–6)	0.129
IADL score (*n* = 45) (median, IQR)	7 (5–8)	6 (2.75–7)	0.116
MOB score (*n* = 44) (median, IQR)	3 (3–3)	3 (2–3)	0.281
SPMSQ score (*n* = 45) (median, IQR)	0 (0–0)	0 (0–1)	0.115
MNA‐SF score (*n* = 45) (median, IQR)	12 (9–13)	11 (9.75–12)	0.122
CIRS‐CI score (*n* = 45) (median, IQR)	4 (3–5)	4 (3–5)	0.888
Number of drugs (*n* = 45) (median, IQR)	8 (6–10)	6 (4.75–8)	0.110^a^
*Cohabitation status (n* = *45) (n, %)*			
With family	17/21 (81%)	17/24 (70.8%)	
Institutionalised	1/21 (4.8%)	1/24 (4.2%)	0.669^b^
Alone	3/21 (14.3%)	6/24 (25%)	
MPI score (*n* = 45) (median, IQR)	0.250 (0.250–0.357)	0.375 (0.313–0.453)	0.040*
SF‐12—physical component (*n* = 39) (mean ± SD)	39.5 ± 9.1	36.3 ± 9.2	0.281^a^
SF‐12—mental component (*n* = 39) (mean ± SD)	47.8 ± 10.5	44.4 ± 8.6	0.272^a^
*Negative health outcomes*			
Institutionalization (*n*, %)	1 (3.3%)	4 (14.3%)	0.138^b^
Re‐hospitalization (*n*, %)	3 (10%)	1 (3.6%)	0.334^b^
Death (*n*, %)	1 (3.3%)	0 (0%)	0.330^b^

^*^ Statistically significant *p* value

^a^Comparison made using *t* test

^b^Comparison using Chi‐square test

*MPI* Multidimensional Prognostic Index, *ADL* Activities of Daily Living, *IADL* Instrumental Activities of Daily Living, *MOB* Barthel Mobility, *SPMSQ* Short Portable Mental State Questionnaire, *MNA–SF* Mini‐Nutritional Assessment–Short Form, *CIRS‐CI* Cumulative Illness Rating Scale—Comorbidity Index

**Fig. 1:**
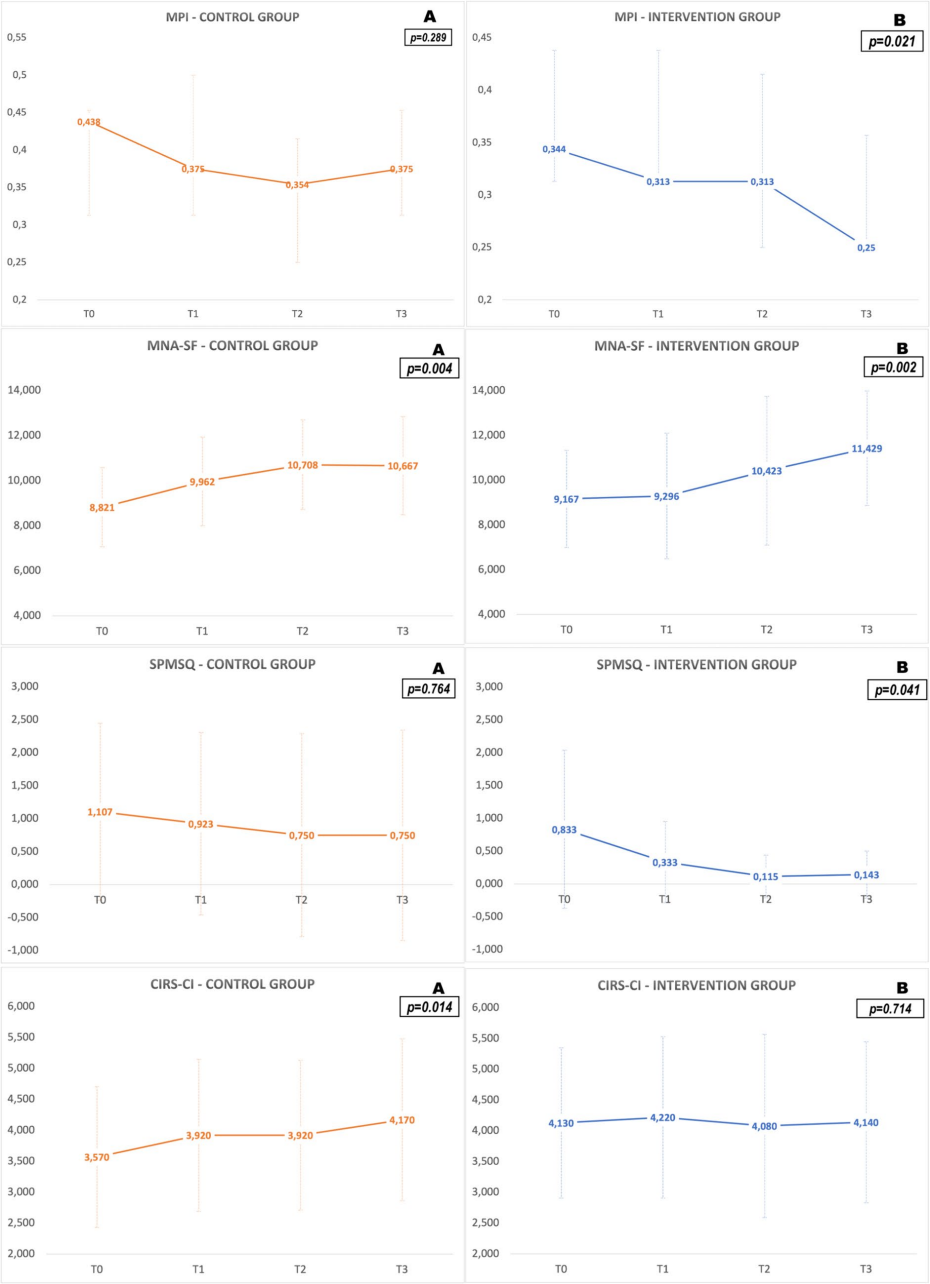
MPI (median scores), MNA‐SF, CIRS‐CI and SPMSQ (mean scores) trajectories over 6 months. The trend of the median (for MPI) or mean scores (for the other tests) and the standard deviations or IQR at the four‐time points assessments divided by the two subgroups (**A** for the Control Group and **B** for the Intervention Group) for the MPI and the three domains which showed a change over time. The *p* values refer to the comparison between the baseline and the third follow‐up. Higher scores in the MNA‐SF mean a better nutritional status whilst lower SPMSQ values suggest a normal cognition

The IG participants showed a significant improvement in multidimensional frailty (MPI score median (IQR) value at T0 = 0.34 (0.31–0.44) vs. T3 = 0.25(0.25–0.36), Wilcoxon’s *W* = 91; *p* = 0.014) (Fig. 1B), nutrition (MNA‐SF score median (IQR) value at T0 = 9.5 (7.25–11) vs. T3 = 12 (9–13), Wilcoxon’s *W* = 12; *p* = 0.002) and cognitive impairment (SPMSQ score median (IQR) value at T0 = 0 (0–2) vs. T3 = 0 (0–0), Wilcoxon’s *W* = 40; *p* = 0.041) (Fig. 1B).

Conversely, CG participants did not show significant changes in the MPI score (Fig. 1A), while a significant worsening of comorbidity (CIRS‐CI score median (IQR) value at T0 = 4 (3–4) vs. T3 = 4 (3–5), Wilcoxon’s *W* = 3.5; *p* = 0.014), an improvement in nutrition (MNA‐SF score median (IQR) value at T0 = 9 (7–10) vs. 11 (9.75–12), Wilcoxon’s *W* = 24; *p* = 0.004) were observed (see Fig. 1A).

Overall, after 6 months from the hospital discharge, IG participants showed a significant improvement in multidimensional frailty than CG participants (MPI median IQR = 0.25(0.25–0.36) vs. 0.38 (0.31–0.45), Mann–Whitney’s *U* = 163; *p* = 0.040).

Regarding SF‐12, no differences were found between the two groups neither for the physical component (SF‐12 physical component score mean value IG = 39.5 ± 9.1 vs. CG = 36.3 ± 9.2; *p* = 0.281) nor for the mental one (SF‐12 mental component score mean value IG = 47.8 ± 10.5 vs. CG = 44.4 ± 8.6; *p* = 0.272).

## Discussion

In this study, we found that a transitional care program based on a multicomponent intervention involving cognitive and physical activation, educational program, and music‐based relaxation activities in a technologically monitored intrahospital environment reduced LOS.

Our multicomponent intervention favored a reduction of approximately 2 days in LOS in the IG, regardless of the subject’s degree of frailty (MPI‐1 and MPI‐2). The importance of this finding is accentuated by the difference found in the literature between younger and older patients, showing an average LOS of 1.4 days longer for the latter ones [4].

The reduction in hospitalization could be beneficial for patients and for public health systems as a whole, allowing staff to experience less pressure and the health system to save resources by having more free hospital beds and fewer patients with hospital‐acquired complications requiring a prolonged or a new hospitalization [3]. Furthermore, the findings of this study can partially fill the gap in the literature about the effectiveness of discharge planning, in‐depth assessment, multidisciplinary care, and telehealth in reducing hospitalization length in high‐risk populations, such as older patients.

Data from this study highlights the urge to plan a care path after discharge to help frail older adults return to pre‐existing levels and recover [37, 38]. In fact, at the moment of the study inclusion, the patients included presented HRQOL values below the cut‐off scores reported in the literature in both the physical (cut‐off = 40.69) and the mental (cut‐off = 51.35) components.

In addition to the set objective, after 6 months the IG's participants showed a significant decrease in multidimensional frailty allowing them to pass from a moderate risk of frailty (MPI‐2; score > 0.33) to a low risk of frailty (MPI‐1; score < 0.34). The CG's participants did not show the same trend as they remained in the MPI‐2 category of risk throughout the follow‐up period. This finding suggests the concept that multidimensional frailty could be a reversible condition [12], in the hospitalized older population. Literature shows that higher frailty levels are associated with a prolonged LOS [39], however, our findings highlight the inverse relationship too: multidimensional frailty can be improved by a reduced LOS.

Along with diminished hospitalization duration, specific aspects of the multicomponent program could have played a role in improving the MPI and specifically the cognitive and nutritional domains. More specifically, the IG's patients showed an improvement in cognitive status which could have been facilitated by the specific cognitive activity program. Both the IG and CG reported an improvement in nutritional status since the discharge. The baseline nutritional assessment demonstrated that older subjects were on average at risk of malnutrition (MNA median score between 8 and 11) likely due to the effects of hospitalization itself. According to previous literature findings, about 30% of patients with a good nutritional status before admission will develop malnutrition during hospital stays [40]. Indeed, a worsening in the nutritional status during hospitalization might be caused by several factors including loss of appetite, fasting for diagnostic procedures, and side effects of medications [41].

Finally, we found that in the CG, there was an increase in the comorbidity index. As at the beginning of the study, the two groups did not show differences, it is possible that the reduced LOS together with the continuous telemonitoring preceding IG’s discharge, could have played a protective role in the IG's participants regarding the development of new comorbidities or decompensation of previous ones.

During the follow‐up, one participant of the IG died due to complications following an accident. This event was judged by the researchers as not related to the PRO‐HOME TCP.

This study is not without limitations. The first is the small number of participants which implies that our encouraging results must be taken cautiously, until further replications and validations. Two participants were excluded from the initial sample as reporting a very high frailty level which was not comparable to the other participants, this prevented us from reaching the fixed number derived from the sample calculation. Additionally, for safety reasons, our sample did not include people at high risk of frailty (MPI‐3).

We encourage future research involving people with a higher level of frailty because we effectively showed both the safety and efficacy of the program in the frailer subjects of our sample. Similarly, further studies on people with behavioral disorders, psychotic symptoms, or moderate/severe impairments in functional autonomy and/or cognition, who were excluded from this trial, should be carried out to generalize the obtained findings.

Another limit is that data on the acceptance and satisfaction of overall TCP were not collected thus preventing from using participants’ feedback to improve future TCPs. Finally, the perception of technological monitoring devices, in terms of technostress [42], was not evaluated. Focusing on the acceptance of technological instruments could guide the selection of the most beneficial and non‐invasive tools to be applied in a protected discharge environment or in a future domiciliary deployment.

## Conclusions

In conclusion, a transitional care program based on a multicomponent intervention involving physical activities, cognitive training, and a lifestyle educational program employing a telemonitoring system inside a protected in‐hospital facility can effectively reduce the length of hospital stay and the multidimensional frailty 6 months after discharge in hospitalized older people.

## Data Availability

The datasets generated and/or analysed during the current study are not publicly available because the project network in which PRO‐HOME is involved is still ongoing thus further analysis will be conducted. However, the datasets are available from the corresponding author on reasonable request.
